# Controversies in the diagnosis and treatment of early cutaneous melanoma

**Published:** 2015

**Authors:** OA Orzan, A Șandru, CR Jecan

**Affiliations:** * “Carol Davila” University of Medicine and Pharmacy, Bucharest, Romania; ** Department of Dermatology, Elias University Emergency Hospital, Bucharest, Romania; *** Department of Surgical Oncology, “Alexandru Trestioreanu” Oncologic Institute, Bucharest, Romania; **** Department of Plastic and Reconstructive Microsurgery, “Prof. Dr. Agrippa Ionescu” Clinical Emergency Hospital, Bucharest, Romania

**Keywords:** early cutaneous melanoma, diagnosis, treatment, follow-up

## Abstract

Cutaneous melanoma (CM) is a disease with an unpredictable evolution mainly due to its high metastatic ability. The steadily increasing incidence and the poor outcome in advanced stages made this cancer an interesting field for many research groups. Given that CM is a curable disease in early stages, efforts have been made to detect it as soon as possible, which led to the diversification and refining of diagnosis methods and therapies. But, as the data from trials have been published, doubts about the indications and efficacy of established treatments have arisen. In fact, there is probably no single aspect of early CM that has not given birth to controversy. This article intends to present the current disputes regarding the early detection, diagnosis, treatment and postoperative follow-up of patients with localized CM. After analyzing both pros and cons, several conclusions were drawn, that reflect our experience in managing patients with early CM.

## Introduction

Cutaneous melanoma (CM), a tumor arising from melanocytes, is one of the most aggressive cancers seen in humans. Its incidence continues to increase worldwide, being one of the most common types of cancers seen in young adults [**1**]. CM represents 3% of all skin cancers, but it is responsible for 65% of the skin cancer deaths [**2**]. However, the early detection and appropriate treatment of the tumor leads to a cure rate of over 90% in patients with incipient melanoma [**3**].

CM can occur anywhere on the skin surface, but its location in a particular segment of the body seems to be influenced by the patient’s sex and age. In men, CM develops most frequently on the back and in women on the lower extremities. Around 20% of all tumors are found in the head and neck area where they have a poorer prognosis than CM at other sites [**4**]. 

A surgical procedure is warranted in all stages of CMM, with better results to any adjuvant therapy. If dissemination has already occurred, surgical treatment must be associated with a form of adjuvant therapy, but in localized CM, outside clinical trials, surgery is perfectly able to provide healing. Therefore, in the multitude of controversies that surround this disease treatment, there is an area of agreement: for patients with stage I and II CM, the mainstay of treatment is a form of surgery. Which one? When should it be applied? Which are the patients it should be applied to? Discussions start from this point.

**Controversies in diagnosis of CM**

**Controversies in clinical diagnosis of CM**

In order to assess the correct treatment and prognosis, it is mandatory to accurately stage the tumor. There are five stages according to the patients’ prognosis: stage 0 (*in situ* melanoma), stages I and II (localized disease), stage III (regional disease) and stage IV (distant metastatic disease).

As approximately 85% of the patients are diagnosed with localized CM (stages I and II) [**5**], the conflicting issues regarding these tumors diagnosis and treatment will be discussed.

Unlike most malignancies, in CM the primary tumor staging is not a clinical one, but a histopathological one, because a correct measurement of tumor thickness, Breslow index respectively, is only possible microscopically. Breslow index stands for the thickness of the tumor measured in millimeters from the granular layer of the epidermis to the deepest level of the tumor penetration and it is unanimously considered the most important factor for survival in the early stages of disease.

Therefore, the aim of the initial biopsy is not only the sampling of a tumor fragment for diagnosis, but also the appreciation of primary tumor (T) characteristics, depending on which subsequent intervention is set.

Four clinical types of CM have been described: superficial spreading melanoma, nodular melanoma, lentigo maligna melanoma and acral lentiginous melanoma. The clinical diagnosis can be achieved by using 2 clinical “rules”: ABCD checklist and “ugly duckling sign”. 

The widely used acronym ABCD, which refers to certain features of the tumor, such as asymmetry (A), irregular border (B), multiple colors (C), a diameter greater than 6 mm (D), has several limitations, such as low specificity despite the high sensitivity and the fact that it does not apply for nodular CM. The second clinical rule, the “ugly duckling sign” refers to the detection of a pigmented lesion that is different from the other lesions in the same individual. 

Based on the clinical evaluation alone, the diagnosis of CM may be highly suspected in a large number of cases, especially in advanced forms of melanoma. However, the clinical diagnosis of incipient CM is still a challenge as it may mimic other benign melanocytic lesions, especially atypical nevi.

**Controversies in dermoscopic diagnosis of CM**

Diagnostic techniques, such as dermoscopy, which is a noninvasive technique, may increase the sensitivity and specificity for the detection of pigmented lesions. In order to facilitate the use of dermoscopy, various diagnostic algorithms have been elaborated. There is not a controversial issue anymore if dermoscopy improves or not the diagnostic accuracy, because this has already been demonstrated by several studies [**6**]. The problem is how useful this tool is for the detection of early CM? In advanced forms of CM, clear-cut features, like the presence of pseudopods, blue-white veil and irregular vessels may be found. However, other features seen in CM, like irregular pigmented network, asymmetry, focal sharply cut-off border, multiple colors, may also be found in atypical nevi, Spitz and Reed nevi.

Consequently, research was focused on identifying those dermoscopic features seen in incipient forms of CM and the initial diagnostic algorithms were revisited in order to increase the sensitivity for CM detection [**7**]. In 2014, at World Congress on Cancer of the Skin, Bowling emphasized the need to focus on the early changes seen in a CM, including a globular reticular network, atypical pigmented network, irregular dots and globules, negative network, regression structures, irregular pigmented streaks, irregular pigmentation [**8**]. 

**Controversies in pathological diagnosis of CM**

The gold standard diagnosis tool for melanocytic tumors remains the histological examination. The method of tissue sampling through different maneuvers, such as punch or shave biopsy, excisional or incisional biopsy, has been widely debated for decades because, as it was already stated, it must provide a sample of tissue that contains the maximum height of the tumor.

Choosing an inadequate biopsy technique can delay or create difficulties in establishing the diagnosis or, in the worst case, can lead to a false negative result.

It seems that the excisional biopsy of the primary tumor with narrow margins (1-3 mm) gained ground in the battle with the other types of biopsy, although without removing them completely from the range of possibilities to establish diagnosis in particular situations [**9**-**11**]. 

**Fig. 1:**
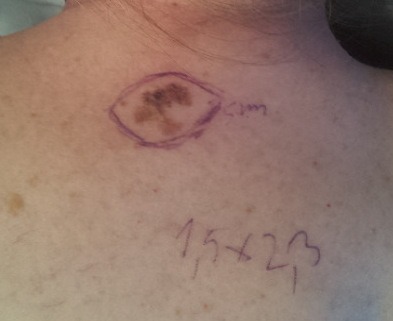
Excision of suspicious lesion with 3 mm margin

The excision orientation should take into account the local lines of tension, as it can be seen in **Fig. 1**, in order to decrease the necessity of skin grafting or extensive reconstructive procedures. A personal approach consists in a round excision with the proposed margins and subsequently the excision of the tegument extensions needed for a linear closure, that reveal themselves clearly under the local tissue tensions.

Large initial excisions or elaborate reconstructions, just for diagnostic, should be resisted, as they may interfere with lymphatic mapping, thus jeopardizing the accuracy of subsequent lymphoscintigraphy, and may also increase the size of subsequent wide local excision for therapeutic purpose.

If tumors are so large that their complete excision would leave a major cosmetic or functional defect, another type of technique should be used such as the punch, incisional or shave biopsy. However, each of these, have several disadvantages, like the risk of missing the malignant changes of the melanocytic lesions and/ or not identifying the real tumor thickness. Besides that, in case of shave biopsies, the process of scarring at the base of biopsy will make malignant cells identification and tumor thickness measurement more difficult [**9**-**12**].

In its various types, incisional biopsy is accepted only in few, clearly defined instances, in which the lesion’s approach by excisional biopsy may be accompanied by higher, unacceptable morbidity [**13**-**17**].

• Pigmented tumors exceeding 2 cm, with particular anatomical locations, whose total excision would result in important cosmetic and functional alterations: face, ears, scalp, hands, plant, subungual region

• Pigmented tumors with low malignant suspicion

• Very large pigmented tumors whose complete excision would result in unreasonably high skin defects in the absence of a clear diagnosis

• Pigmentary giant nevus with a recently macroscopically modified area

The main reproach against incisional biopsy refers to the quite high risk, in some statistics ranging between 16-43%, of T element under staging, which deprives the patient of the appropriate therapy according to his real stage [**15**,**18**,**19**].

However, the over staging is excluded in tumors that extend along skin appendages, the diagnosis being difficult in a small tumor fragment, and thus the patient is exposed to an unnecessarily aggressive treatment [**18**].

Cutting only a fragment, obviously, from the macroscopically most suspected of malignancy tumor region (the most pigmented and most irregular part), incisional biopsy sometimes fails to provide the pathologist the piece with the maximum thickness and thus prevents the accurate assessment of the primary tumor and leads to patient assignment to a more favorable prognosis group [**14**,**19**,**20**].

Although it seems unlikely that a partial removal of a tumor does not affect the patient’s subsequent evolution, however, more published studies state that an incomplete excision of a CM for diagnosis, does not adversely influence either the overall survival or disease free survival [**10**,**11**,**21**,**22**],

This finding could be explained by the fact that, in most cases, the complete excision of the residual lesion is achieved within a relatively short time after the first intervention (2-3 months), which theoretically would prevent a possible dissemination [**23**,**24**].

Although incisional biopsies are no longer blamed, as they were 20 years ago, current guidelines state that excisional biopsy is the gold standard in CM diagnosis [**13**]. There are consistent data that show there are no real differences in prognosis, recurrence risk, disease-free interval or overall survival of melanoma patients, according to the type of biopsy performed [**25**,**26**]. However, surgical common sense make the surgeon feel that only through a full-thickness excision of the suspicious lesion, the pathologist can assess the true depth of the lesion and thus achieve an accurate staging.

Mohs surgery was proposed for the oncologic excision of CM. Nevertheless, frozen sections, inherent to the advocated technique, may induce difficulties in melanic cells identification, which make it unusable in this particular situation [**27**].

The histologic differential diagnosis between benign and malign melanocytic lesions is often challenging. In some cases, immunohistochemistry may be useful. 

An important feature found in CM is the pagetoid spread of atypical melanocytes into the epidermis, but this feature is not considered diagnostic for melanoma anymore as it may be seen in several types of nevi (Spitz, acral, vulvar, etc.) [**28**].

There is a group of borderline melanocytic lesions that cannot be clearly defined as benign or malign. These lesions have a biologically indeterminate behavior, they may reoccur after the excision and they may even metastasize. The borderline melanocytic lesions may be divided in superficial melanocytic proliferations of uncertain significance (SAMPUS) [**29**] and melanocytic tumors with indeterminate biological behavior (of uncertain malignant potential) (MELTUMPS) [**30**,**31**].

SAMPUS should be differentiated by *in situ* and superficial forms of CM and they are characterized by atypical melanocytes found in the epidermis or at the dermo-epidermal junction.

MELTUMPS have a clinical behavior difficult to predict and they should be differentiated by CM arising in a nevus, nodular CM, spitzoid CM, as well as several types of nevi (Spitz, Reed, blues nevus, etc.) [**30**,**31**].

**Wide excision of primary tumor**

Even after CM diagnosis, the multiple-choice questions have not been excluded. What interval from the minimum diagnostic biopsy should wide excision be performed at? What does excision with oncological safety margins mean? If, for some of these questions, answers that have been supported by most authors were found, for other questions we are still looking for responses. A few decades ago, the proposed safety margins, independent of tumor thickness, were standardized to 4 to 5 cm. The substantiation of these margins is probably based on anecdotal reports of singular cases [**32**].

Following CM diagnosis by one of the biopsy techniques described above and detailed microscopic analysis of the resected piece, current guidelines recommend postoperative scar excision with a rim of healthy tissue directly proportional to the primary tumor Breslow index [**13**-**16**,**19**,**33**,**34**]. This reintervention is considered mandatory, although there are two large pathological studies that have shown that the presence of significant residual tumor tissue is unlikely to appear in the absence of its macroscopic identification in the whole specimen [**35**,**36**].

**Fig. 2:**
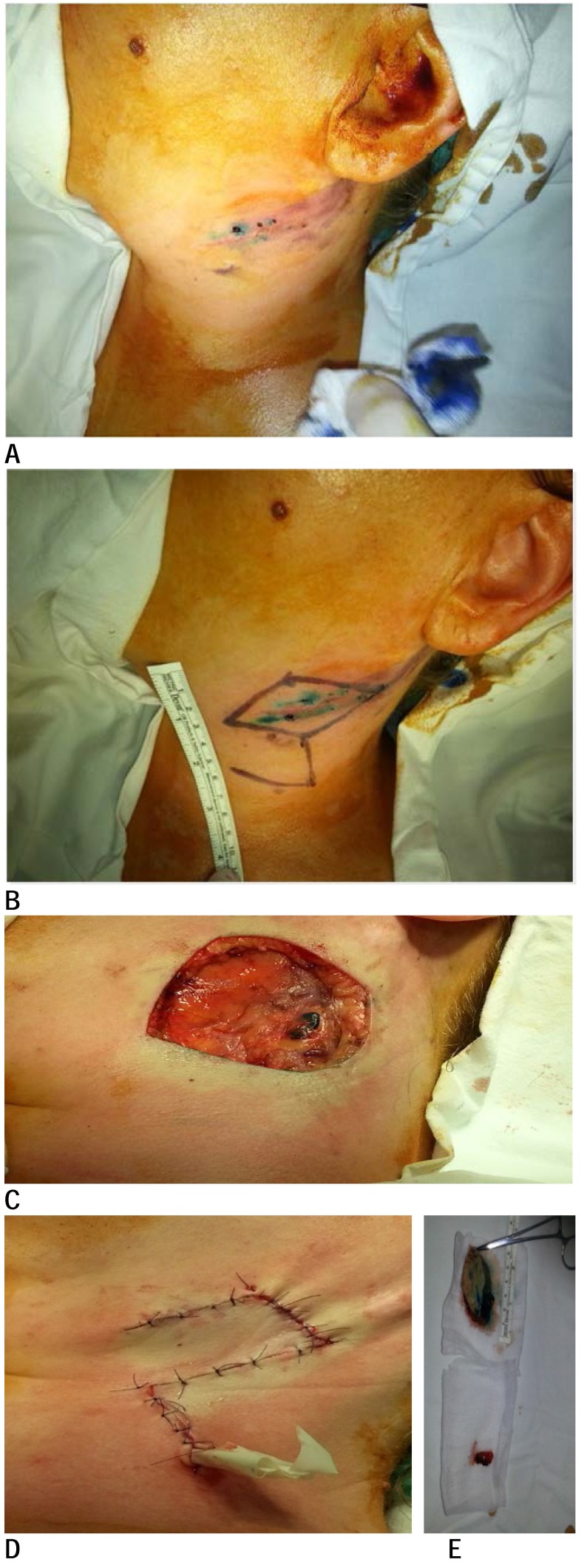
A 33-year-old woman with melanoma of the neck with a Breslow depth of 2,4 mm. (A) Site after initial excisional biopsy, (B) a 20 mm margin wide excision, (C) local defect after excision and sentinel lymph node, (D) Limberg fasciocutaneous flap for reconstruction, (E) wide excision and lymph node specimen

Then, in this context, what is the purpose of this wide resection of the excisional biopsy scar in case of a tumor that proved to be CM? Is this 2-steps procedure recommended or is it excessive?

A retrospective study of the Scottish Melanoma Group showed without any doubt that local recurrence and distant disease free survival, as well as the overall survival were significantly higher in those patients who had a surgical reintervention after diagnosis of CM [**37**].

The definition of oncological safety margin for melanic tumors underwent multiple adjustments over time depending on the progress of knowledge regarding the dissemination of this neoplasia. Historical data stipulated the ablation of at least 5 cm of macroscopically free of disease peritumoral tissue, but the compliance with this rule led to the occurrence of a skin defect almost impossible to suture per primam. These large resections were motivated by their advocates, by highlighting a large number of atypical melanocytes in the skin adjacent to the tumor, up to a distance of 4-5 cm [**38**].

In order to define the notion of oncological safety margin based on scientific data, several randomized clinical trials have been initiated after the seventies. They aimed to determine the optimal distance between the macroscopic edge of the primary tumor and the surgical incision that would provide a low local recurrence rate with a minimal associated morbidity.

Most publications point towards the following recommendations regarding the size of healthy peritumoral tissue that must be excised, depending on the depth of tumor invasion, as it can be seen in **Table 1** [**9**,**13**,**14**,**16**,**33**,**34**,**39**]:

**Table 1:** 

Lesion	Recommended margin
Precursor lesions	Complete excisional biopsy with 1-3 mm
In situ	5 mm
Breslow < 1.0 mm	10-20 mm
Breslow between 1-2 mm	10-20 mm
Breslow between 2-4 mm	20 mm
Breslow > 4 mm	20-30 mm
Unfavorable prognosis (neurotropism, desmoplastic CM, satellite lesions)	30 mm or more

But in all aforementioned studies, only Breslow index was taken into account for setting optimal resection margin, which we believe is not enough. Why ignore tumor biology? Why skip the other features of the primary tumor, such as the presence of lymphovascular invasion or the tumor location or melanoma subtype, factors known to influence the risk of local recurrence? [**40**,**41**].

Special conditions, such as desmoplastic melanoma, neurotropism, satellitosis may necessitate an excisional margin of more than 3 cm [**42**].

But, even if we consider only the Breslow index, recent meta-analysis findings question the safety of narrow excision margins listed above [**43**,**44**]. Furthermore, for thick CM, defined as having a Breslow index greater than 4 mm, the United Kingdom Melanoma Study Group believes that a resection margin of less than 3 cm is accompanied by several local recurrences and a higher risk of death [**45**]. And it looks like we went back to where we started!

Another topic that has sparked controversy concerns the optimal timeframe between the diagnostic biopsy and the wide resection with curative intent. Does the time elapsed between the 2 procedures influence further patients survival? The answer seems to be No: there is no evidence that the overall and disease free survival are influenced by the time interval between the 2 surgeries, as long as it does not exceed 90 days [**24**,**33**].

Although there are several authorized voices who claim that, based on current data, the optimal excision edge for CM cannot be determined [**43**,**44**], until further conclusive data, existing guidelines should be applied, noting that the treatment should be individualized for each patient.

**Sentinel lymph node biopsy**

After analyzing the evolution of over 30 thousands of patients with stage I-III CM, in the last AJCC staging system from 2009, it is specified that the disease prognosis largely depends on two factors [**5**]:

• Primary tumor thickness (depending on which resection limits are set)

• The presence or absence of metastases in regional lymph nodes

In the absence of clinical or paraclinical detectable metastases in the regional lymph nodes, their pathological status can be assessed by the sentinel lymph node biopsy (SLNB) technique introduced in 1992 by D. Morton.

Designed both as a staging tool, but also as a treatment one, if the sentinel node (SN) turns out to be invaded by neoplastic cells, this complex intervention that involves a close collaboration between a nuclear medicine physician, pathologist and surgeon, was the subject of several clinical studies, of which perhaps MSLT1 (Multicenter Selective Trial Limphadenectomy 1) is the largest. This trial aimed to prove that SLNB achieves a correct staging of regional lymph node basins, increases disease regional control by carrying out a completion lymph node dissection (CLND) when a positive SN is detected and improves survival [**46**,**47**].

Although SLNB is considered standard in most therapeutic guidelines adopted by different professional associations [**48**,**49**], as data from multicenter trials grow, the usefulness of this procedure has been questioned [**50**,**51**]. What is an issue, is its usefulness as a treatment method, because, until now, no one challenged its value as a mean of regional lymph nodes staging.

The theoretical model from which Morton et al. started the development of the selective lymphadenectomy concept, was based on the hypothesis that CM invades lymphatic vessels first, in an orderly manner and only later, after the invasion of regional lymph nodes occured, spreads to distant organs.

According to this hypothesis, called “Incubator hypothesis”, cancer cells detached from the primary tumor get through afferent lymphatic channels in a first node (named sentinel node), where they multiply, acquire metastatic properties and only afterwards invade other lymph nodes from the regional basin [**52**,**53**]. It has been postulated and subsequently demonstrated that the pathological status of the sentinel lymph node reflects the status of the entire lymph node basin [**52**].

But the disputes have arisen right away [**54**,**55**]. Why would melanic cells strictly follow this way to metastasize? What prevented them to spread hematogenously first? Or why would not lymphatic and hematogenous metastases develop simultaneously? Once the questions have emerged, the theories that have attempted to explain the observed phenomena have also arised. And perhaps, the most confusing finding was the discovery of a distant metastasis in the event of negative sentinel nodes. 

Supporters of the “marker hypothesis” believe that melanocytes simultaneously disseminate via lymphatic and hematogenous pathways and regional lymph node invasion is just an indisputable sign of distant metastasis [**37**]. However, in our experience, this statement is not always true.

From our point of view, the most complete and thruthful hypothesis is that issued by Pizzaro. Called differentiatial spread pattern, this hypothesis divides CM into 4 groups according to their metastatic potential. According to this theory, only around 30% of CM patients are qualified for SLNB, namely those patients whose tumors metastasize exclusively by lymphatics [**55**]. But, so far, there has been no evidence (clinical, histological or molecular) that allowed us to guess the metastatic paths of each individual CM. We only have an overall picture.

Is SLNB mandatory for the assessment of the status of regional lymphatic basin? Could it be replaced by a less aggressive investigation like ultrasound of regional lymph nodes followed by fine needle aspiration (FNAC) of the suspected ones? Which patients SLNB is addressed to?

Most authors consider that SLNB should be recommended to all patients with stage I and II CM who have an estimated risk of metastasis in the regional lymph nodes of at least 10% [**5**,**56**]. The magnitude of risk varies in different studies depending on several factors: primary tumor thickness, presence or absence of ulceration, mitotic index, the patient’s age [**57**] and the list can go on.

It should be noted that in at least one regard, consensus has been reached: patients with intermediate CM, ie Breslow index between 1-4 mm, should be unreservedly recommended the SLNB procedure. The above directive was supported by the fact that in this patient population, the risk of occult metastases in the regional lymph nodes is large enough, around 15-30% [**46**], to justify the intervention, and at the same time, the probability of having distant metastases is sufficiently low so that the maneouver would not become redundant.

Regarding thin CM, ie Breslow index < 1 mm, and thick CM, ie Breslow index > 4mm, opinions are divided and guidelines continue to evolve.

A CM with a depth of invasion of less than 1 mm has a quite small probability of regional lymph nodes metastases at diagnosis, somewhere between 1-5% [**58**,**59**]. Therefore, in this group of patients, SLNB should be taken into consideration only if there is clear evidence of an increased risk of dissemination based on tumor biology.

The multivariate logistic analysis was used to study the impact of primary tumor ulceration, Clark level of invasion, mitotic index, regression, lymphovascular space invasion (LVSI) and tumor infiltrating lymphocytes (TILs), on the risk of metastasis in the regional lymph nodes. After analyzing all these factors, three categories of tumors with different metastatic ability were distinguished into the thin CM group. Fortunately, the group with the highest risk of metastasis represents only 3.5% of this population, but the 10-year mortality rate for this category, is significantly higher than for the others, around 15.6% [**60**].

As most studies have shown that among thin CM, tumors with Breslow index greater than 0.75 mm are responsible for 86% of SLN metastasis [**59**], the following consensus has been reached: young patients with ulcerated tumors, Breslow index between 0.75 to 1 mm, or with more than 1 mitosis/ mm2, or with lymphovascular space invasion, or Clark level IV or V, have a risk of occult lymph node metastases greater than 5% and therefore can and should benefit from SLNB.

If in thin CM reluctance to the method comes from the fact that it involves too many investments for the discovery of a relatively small number of patients who could truly benefit from SLNB, in case of thick CM identification of lymph node metastases, often coincides with systemic metastases, so in this situation also, the procedure brings advantages only to a small number of patients.

Thick CM have a high metastatic capacity [**5**,**61**]:

• 35-45% of newly diagnosed tumors already have metastases in the regional lymph nodes 

• 3 of 4 cases develop systemic metastases during subsequent evolution

• 10 years overall survival rate ranges between 50 and 68%

But for thick CM also, SN status is the most important independent prognostic factor for survival [**30**,**45**]. Therefore, ASCO (American Society of Clinical Oncology) and SSO (Society of Surgical Oncology) believe that SLNB should be recommended for all the patients with thick CM both as a staging method, as well as to facilitate the regional control of the disease if SN is positive. SLNB allows the stratification of patients with thick CM and their subsequent enrollment in clinical trials [**49**,**62**].

Another controversial issue is the sequence between SLNB and the wide excision of the primary tumor. There are authors who consider that SLNB has a maximum accuracy if it is carried out during the same procedure with the primary tumor excision [**63**]. Otherwise, the disruption of afferent lymphatic vessels during the first surgery can cause a distortion of the lymphatic drainage with the appearance of aberrant pathways [**64**]. And so, there is the risk that the lymphatic path visualized after the radiotracer injection around the postoperative scar does not overlap with the initial lymphatic drainage of the primary tumor [**65**].

Unfortunately, this goal is not always achievable because, in many cases, the excisional biopsy for a suspicious melanic lesion is done in any medical facility and only later, after receiving the pathological report, the patients are guided to a reference center.

The study carried out by Gannon et al. showed that performing SLNB after primary tumor resection for diagnostic purposes is perfectly feasible and does not affect the accuracy of the method [**66**]. However, some situations in which SLNB has a significant risk of error have been described and therefore separate analysis of these cases is recommended [**56**,**66**]:

• A very wide excision requiring skin grafts or rotation flaps for skin closure; either of these two techniques could applying of either of these two techniques could alter lymphatic drainage

• Previous surgeries for other conditions in regional lymphatic basin

• History of radiotherapy on a field which included regional lymph nodes 

• Acute infection of the remaining wound after primary tumor resection

Perhaps the hottest topic of the moment on SLNB, refers to the role of completion lymphadenectomy. The results of the two trials (MSLT2, MINITUB) that aimed to clarify this matter are not yet available, so the disputes continue.

The proponents of SLNB and current guidelines also recommend that the identification of a positive SN must be followed by a completion lymphadenectomy (CLND) arguing that in this way, the risk of regional relapse and distant dissemination, as the risk of death are reduced [**46**,**53**]. Very likely, but the benefit is obviously only for a small percentage of patients, compared to the large number to which the surgery is done.

Studies published so far claim that from the group of patients with positive SN, only a minority, between 9-25%, have other metastatic regional lymph nodes (non-sentinel positive lymph nodes) [**56**,**67**]. And, considering these circumstances, isn’t the indication to practice routine CLND in all patients with positive SN, overstated? This is because in about 80% of the patients undergoing this invasive surgery, it is not only useless, but also harmful.

Although there are dozens, maybe hundreds of papers, which try to establish a link between the characteristics of the SN neoplastic deposit (subcapsular, central or multifocal location, the maximum diameter of the largest metastatic focus or SN tumor burden as measured by Rotterdam criteria) and the probability of metastasis in non-sentinel lymph nodes, there is no model to guide us to date [**68**-**71**]. 

Researchers from the University of Rotterdam consider that patients with SN metastases of less than 0.1 mm, have the same prognosis with the patients with negative SN and therefore, lymphadenectomy is unlikely to bring them any benefit [**72**]. In this particular situation, ultrasound of lymphatic basin may represent an alternative to lymphadenectomy.

From a variety of conflicting data, it is difficult to draw a reasonable conclusion. Yet, despite the reproofs, we believe that SLNB is an excellent staging method, that SN status still represents the most important prognostic factor for disease free survival and overall survival and, for patients with positive SN, completion lymphadenectomy significantly improves survival.

**General principles of reconstruction**

The wide excision of CM results in defects that can be reconstructed by undermining and primary closure of skin, using in advantage local skin tension lines, by skin grafts and by local or distant flaps. These principles are well known but several questions are still being asked: primary closure or a more elaborated reconstruction, skin grafts or flaps? Should a specific lesion inherit the area specificity or should it be treated as a usual CM? Should the reconstructive ladder be climbed systematically, or should we use the elevator in our advantage?

In real life, many individualized clinical cases require the special skills of a plastic and reconstructive surgeon. There is no room for “one size fits all” paradigm! Several aspects like tumor location, age, skin laxity, patient preference should be taken in account. In our opinion, one should use in advantage the relaxed skin tension lines and acknowledge the utility of various local flaps in specific anatomic sites. 

**Cutaneous melanoma management in special locations**

**Ear.** Appropriate margins according to the tumor thickness, routinely 0,5 to 2 cm, must be respected. Full-thickness wedge excisions, including the cartilage, are indicated in helical rim lesions. The defect can be closed through primary suture, resulting a smaller but normal ear. More elaborated techniques, such as helical rim advancement can be used in larger lesions. CM on the sites of the ear can be excised full-thickness, including the cartilage as the deep margin and skin grafting the dermis of the opposite site. Larger or multifocal lesions can be managed by total or subtotal ear amputation. A real benefit can be obtained for patients wearing glasses by the conservation of the upper part of the ear. 

**Fingers and Toes.** Functional considerations are to be followed in CM of fingers or toes. A balance between the functioning and surgical adequate margins must be sought. Newly pigmented atraumatic streaks of the nail should always be biopsied [**42**]. Bone removal makes no difference unless it is directly involved. Lesions involving the distal phalanx can be managed by amputation proximal to the distal interphalangeal articulation, closing the stump with volar or dorsal classic flaps.

CM located in proximal phalanx can be managed by excision and defect reconstruction with cross-finger flaps, neurovascular flaps or skin grafts. Toes melanomas are treated by metacarpophalangeal disarticulation, taking care to preserve the first and fifth metacarpal head, very important for the normal gait. Rarely is ray amputation advocated, even in web space involvement, when a local flap may be sufficient. 

**Foot.** This region is limited in the local skin resources, so the resulting defect needs some form of reconstruction. For dorsal or non-weight bearing areas thick skin grafts may suffice. For weight bearing area of the sole, even local flaps like toe-filet flap (fore foot) or free microsurgical flap transfer works fine (heel area - fasciocutaneous or muscle skin grafted flaps).

**Breast.** This region should be treated as a normal area, with the margins of excision dictated by CM thickness. Breast amputation is not indicated. In the advent of nipple-areola complex involvement, several techniques are available for later reconstruction. 

**Genital and Anorectal Region.** The genital area is treated same as above, as all the other CM. Unless direct involvement dictated by the pathologist is reported, there is little indication for radical vulvectomy or radical inguinal dissection [**73**]. Anorectal melanoma has a high mortality due to the early metastatic disease. The defect with tumor free margins must be reconstructed with local flaps. Sphincter reconstruction is rarely necessary, unless classic abdominoperineal resection is indicated. 

**Mucosal melanoma.** Mucosal melanoma may arise in respiratory, alimentary and genitourinary tracts and represents only 1% of all melanomas, with a site-specific survival rates at 1, 5 and 10 years, of 80%, 29% and 15%, respectively [**74**]. The treatment of mucosal melanoma with a wide surgical excision of local tumor and regional nodes should be conducted according to the clinical involvement [**73**].

**Follow-up and surveillance**

Follow-up should be differentiated between stage I to IIA disease and stage IIB or IIC patients [**2**].

For stage I to IIA group, anamnesis and physical examination should be done every three months for the first year, every 6 months for the next 4 years and annually thereafter. This raises the question what should the follow-up protocol include, which regions should be checked? The primary excision site, the rest of the skin and regional nodal basin must be assessed. There are no specific laboratory or imaging studies recommended [**75**]. Despite this, many clinicians routinely order chest radiographs, blood counts, liver function tests, serum tumor markers and computed tomography scans. Positron emission tomography–computed tomography (PET/ CT) gained some popularity as it can help identifying small metastases and bring information regarding the surgical resection [**76**]. 

For the second group, stage IIB and IIC, there is some indication for biannually or annually chest Rx, computed tomography or PET/ CT [**77**]. Annual magnetic resonance imaging of the brain may be recommended, but after 5 years any of the aforementioned imaging studies are not useful anymore [**2**].

## Conclusion

Cutaneous melanoma is becoming epidemic and its incidence is expected to increase. Some well-designed trials have changed the management of CM patient and it is expected that the results of ongoing ones will also bring major changes. Although multidisciplinary approach represents the best standard of care for melanoma patients, surgery remains the best option for most localized cases. Early detection, state of the art biopsy and wide local excision according to rules established by clinical trials can cure the disease. SLNB can identify high-risk patients to whom the opportunity of attending clinical trials may be given. We hope that the future will belong to personalized therapy.

**Disclosures:** authors declare no potential conflict of interests

**Acknowledgement:** The authors have equally contributed to this paper editing.

**Sources of Funding:** This paper was co-financed from the European Social Fund, through the Sectorial Operational Programme Human Resources Development 2007-2013, project number POSDRU/159/1.5/S/138907 “Excellence in scientific interdisciplinary research, doctoral and postdoctoral, in the economic, social and medical fields –EXCELIS”, coordinator The Bucharest University of Economic Studies.
